# Glucose-6-Phosphate Isomerase (G6PI) Mediates Hypoxia-Induced Angiogenesis in Rheumatoid Arthritis

**DOI:** 10.1038/srep40274

**Published:** 2017-01-09

**Authors:** Ying Lu, Shan-Shan Yu, Ming Zong, Sha-Sha Fan, Tian-Bao Lu, Ru-Han Gong, Li-Shan Sun, Lie-Ying Fan

**Affiliations:** 1 Department of Clinical Laboratory, Shanghai East Hospital, School of Medicine, Tongji University, 150 Ji Mo Road, Shanghai 200120, People’s Republic of China

## Abstract

The higher level of Glucose-6-phosphate isomerase (G6PI) has been found in both synovial tissue and synovial fluid of rheumatoid arthritis (RA) patients, while the function of G6PI in RA remains unclear. Herein we found the enrichment of G6PI in microvascular endothelial cells of synovial tissue in RA patients, where a 3% O_2_ hypoxia environment has been identified. In order to determine the correlation between the high G6PI level and the low oxygen concentration in RA, a hypoxia condition (~3% O_2_) *in vitro* was applied to mimic the RA environment *in vivo*. Hypoxia promoted cellular proliferation of rheumatoid arthritis synovial fibroblasts (RASFs), and induced cell migration and angiogenic tube formation of human dermal microvascular endothelial cells (HDMECs), which were accompanied with the increased expression of G6PI and HIF-1α. Through application of G6PI loss-of-function assays, we confirmed the requirement of G6PI expression for those hypoxia-induced phenotype in RA. In addition, we demonstrated for the first time that G6PI plays key roles in regulating VEGF secretion from RASFs to regulate the hypoxia-induced angiogenesis in RA. Taken together, we demonstrated a novel pathway regulating hypoxia-induced angiogenesis in RA mediated by G6PI.

Rheumatoid arthritis (RA) is an auto-immune disease characterized by excessive proliferation of synovial tissue, inflammation in the joints and formation of capillary^1^,^2^. RA synovium contains high levels of inflammatory cytokines and enzymes, leading to degradation of articular cartilage and subchondral bone^3^.

Glucose-6-phosphate isomerase (G6PI) plays a crucial role in glycolysis and gluconeogenesis through catalyzing the interconversion of D-glucose-6-phosphate and D-fructose-6-phosphate^4^,^5^. Furthermore, G6PI can be secreted to the outside of cells functioning like a cytokine or growth factor^6^,^7^. In RA patients, the levels of G6PI including soluble G6PI and G6PI immune complex are significantly higher in both sera and synovial fluid^8^. Recombinant G6PI is able to induce chronic arthritis in mouse model, resulting RA-like systemic and/or distal arthritis^9^.

Angiogenesis starts at the early phase of inflammation until the formation of new capillaries from the pre-existing vasculature. It has been well demonstrated that the initiation and progression of arthritis are closely related to angiogenesis^10^. Angiogenesis occurs frequently in the inflamed joint^11^. Hyperplasia of RASFs leads to over-proliferation of synovial tissue resulting in increased oxygen consumption in synovium, thereby forming a hypoxic environment. The reduced oxygen level in the synovium of arthritis has been demonstrated^12^. 3% of oxygen level has been confirmed to represent the joint environment in RA^13^. Furthermore, the hypoxia level in inflamed joint is inversely correlated with the levels of vascularity, oxidative damage and synovial inflammation^14^,^15^. HIF-1α, a key gene related to hypoxia, is highly expressed in the synovial tissue^16^. The upregulation of vascular endothelial growth factor (VEGF), angiopoietins, monocyte chemotactic protein 1, interleukin-8, CCL20 and matrix metalloproteinases (MMPs) and down-regulation of interleukin-10 have been reported in synovial cells under hypoxia condition^17^. All of these growth factors and chemokines can regulate angiogenesis.

G6PI is identified having similar function as autocrine motility factor (AMF)^18^, a multifunctional cytokine protein capable of regulating cell migration, invasion, proliferation and survival^19^,^20^. Our previous work has demonstrated that G6PI could increase cellular proliferation and inhibit cell apoptosis in fibroblast-like synoviocytes in RA via promoting G1/S transition of the cell cycle^21^. Literature shows that AMF induces angiogenesis in cancer by increasing the cell motility and the expression of vascular endothelial growth factor receptor (VEGFR) in endothelial cells^22^,^23^,^24^. However, the function of G6PI in RA, and the relationships between hypoxia, G6PI and angiogenesis remain unclear.

In this study, the increased G6PI level was confirmed in RA. We further demonstrated that hypoxia is able to induce angiogenesis and increase the expression of G6PI in both HDMECs and RASFs. By gene loss-of-function assays, we demonstrated the hypoxia-induced angiogenesis is dependent on the G6PI expression in HDMECs and VEGF secretion from RASFs, the latter is also regulated by G6PI.

## Results

### High Expression of G6PI in RA synovial tissue

**Figure 1:**
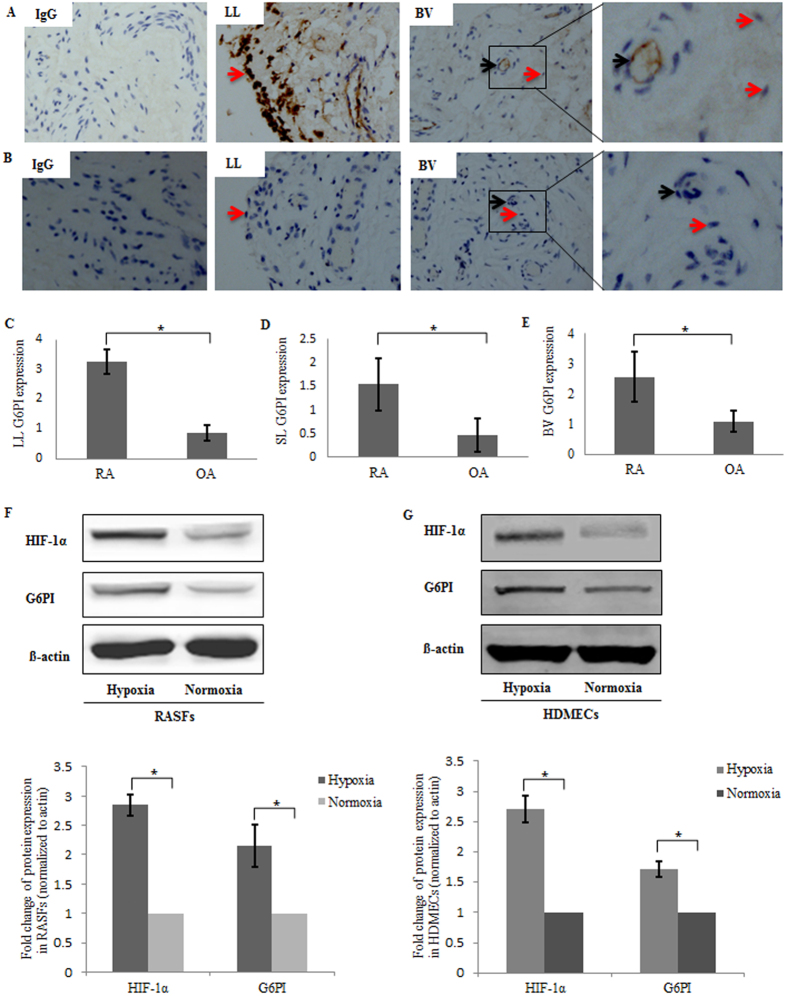
Representative photomicrographs showing G6PI localization in synovial tissue samples from patients with rheumatoid arthritis (RA) and osteoarthritis (OA). (**A**) Immunohistochemical staining of control IgG and G6PI in synovial tissue samples from RA patients (n = 10). Strong G6PI signals were detected around the blood vessels (black arrows) and in the synovial fibroblasts (red arrows) from lining layer, sublining layer. BV = blood vessels. LL = lining layer, SL = sublining layer. (**B**) Weak signals of G6PI was observed in the synovial tissues of OA (n = 10). (**C**,**D**,**E**) G6PI expression in synovium from lining layer (**C**), sublining layer (**D**) vascular region (**E**) of the patients with RA and OA. (**F**,**G**) Representative images showing the expression of G6PI and HIF-1α and quantifications of western blots in rheumatoid arthritis synovial fibroblasts (**F**) and HDMECs (**G**). Data were presented as mean ± SEM. *P < 0.05.

Immunohistochemistry analysis was performed in synovial tissue sections from patients with RA (n = 10) and OA (n = 10) using anti-G6PI. High levels of G6PI were detected in the synovial lining, sublining layers and vascular regions (Fig. 1A–E). Strong G6PI signals were detected around the blood vessels (black arrows) and in the synovial fibroblasts (red arrows) (Fig. 1A), where the oxygen level is as low as 3% under hypoxia condition^13^. Much less expression of G6PI was observed in the synovial tissues of OA (Fig. 1B), compared to RA.

In order to determine the relationship between the G6PI levels and hypoxia, primary RASFs and HDMECs were cultured under 3% O_2_ of hypoxia condition. Western blot analysis indicated the induction of G6PI expression in both RASFs and HDMECs by hypoxia (Fig. 1F and G). As positive control of hypoxia, HIF-1α showed induction by incubation under 3% O_2_ condition.

### G6PI expression is required for the Hypoxia-induced cellular proliferation in RASFs

**Figure 2:**
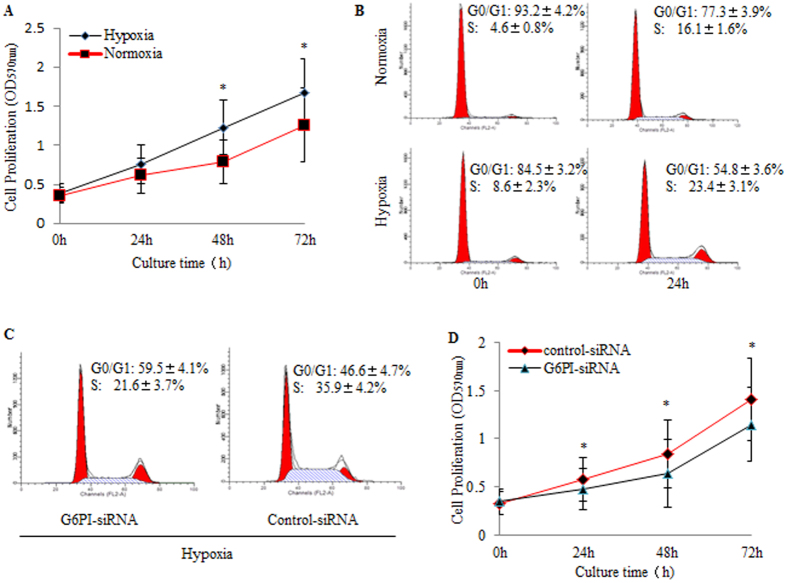
The induced cellular proliferation of RASFs by hypoxia and G6PI. (**A**) The growth curve of RASFs cultured under normal and hypoxia conditions, which were determined by MTT assay. (**B**) The cell cycle analysis by Flow cytometry indicating the increased G_1_/S transition of RASFs under hypoxia condition. (**C**) G6PI-siRNA arrested G_1_/S transition of RASFs. (**D**) G6PI-siRNA suppressed cellular proliferation of RASFs under hypoxia condition. Data were presented as mean ± SEM(n = 3). *P < 0.05.

In order to determine the effect of hypoxia on RASFs, cell proliferation assay and cell cycle analysis were performed under normal and hypoxia conditions. As shown in Fig. 2A, hypoxia promoted RASF cell proliferation *in vitro*. Cell cycle analysis indicated the promoted G_1_/S transition of RASFs under hypoxia condition (Fig. 2B). Interestingly, knockdown of G6PI attenuated the G_1_/S transition promotion by hypoxia, indicating the requirement of the G6PI expression for the hypoxia-induced cell cycle in RASFs (Fig. 2C). Since hypoxia induced the expression of G6PI (Fig. 1F), we further examined the function of G6PI in RASFs by MTT assays indicating the decrease of cell proliferation after treatment with G6PI siRNA in RASFs (Fig. 2D).

### The induction of endothelial cell tube formation by hypoxia requires G6PI and RASFs

**Figure 3:**
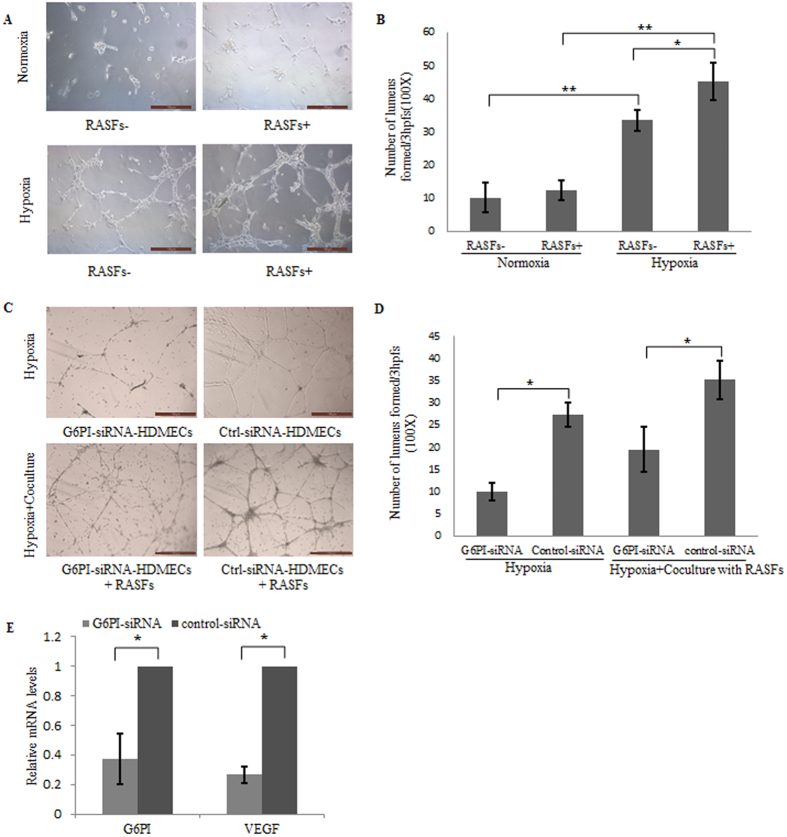
Hypoxia induction of endothelial cell tube formation requiring G6PI and RASFs. (**A**) Representative images showing the induced tube formation of HDMECs under normal and hypoxia conditions, co-culturing with or without RASFs. (**B**) Quantification of A. (**C**) Tube formation of HDMECs treated with G6PI-siRNA or control-siRNA, co-culturing with or without RASFs under hypoxia condition. (**D**) Quantification of C. (**E**) Decreased expression of VEGF mRNA in HDMECs treated with G6PI-siRNA under the condition of hypoxia. Data were presented as mean ± SEM(n = 3). *P < 0.05.

In order to determine the effect of hypoxia on angiogenesis, HDMECs tube formation assays were performed under normal and hypoxia conditions with or without the presence of RASFs. As shown in Fig. 3A, hypoxia induced the tube formation of endothelial cells significantly. By co-culturing with RASFs, HDMECs showed much more tube formation than HDMECs only under hypoxia condition (Fig. 3A,B).

In order to further demonstrate the role G6PI plays in endothelial cells during angiogenesis, G6PI siRNA was transfected into HDMECs followed by tube formation assays. As shown in Fig. 3C, and D knockdown of G6PI decreased tube formation of HDMECs in the presence or absence of RASFs under hypoxia condition. Co-culturing with RASFs clearly increased tube formation of HDMECs.

In order to clarify the mechanism by which G6PI regulates angiogenesis in endothelial cells, VEGF level in the cells was analyzed in HDMECs following treatment with G6PI siRNA or control siRNA. As showed in Fig. 3E, the mRNA level of VEGF in endothelial cells decreased after treatment with G6PI siRNA.

Taken together, both G6PI expression and RASFs co-culture are required for the hypoxia-induced angiogenesis in **endothelial cells**.

### The induction of endothelial cell migration by hypoxia requires G6PI and RASFs

**Figure 4:**
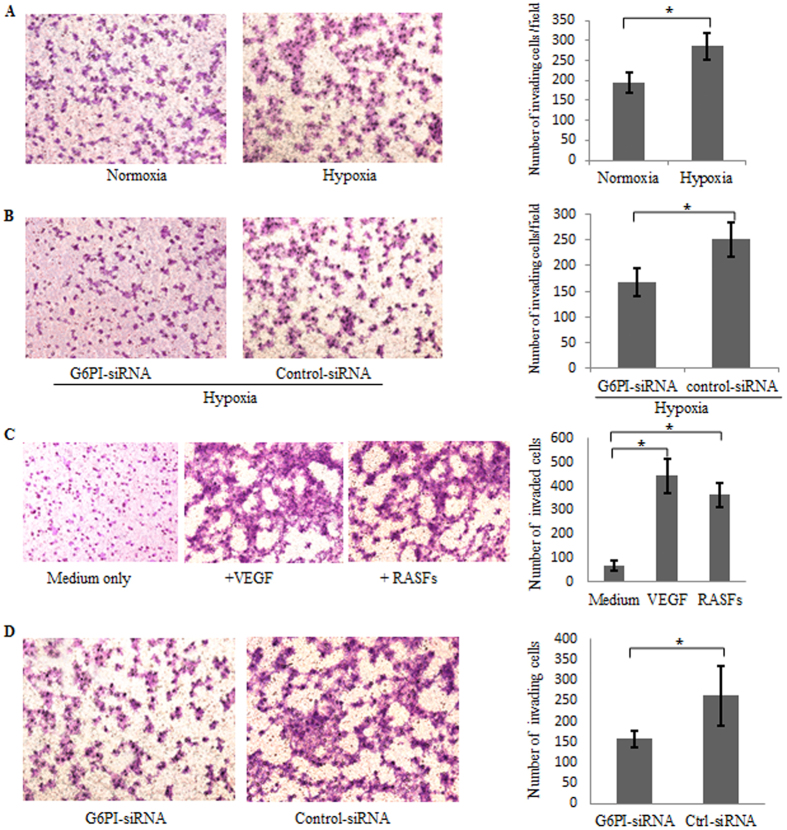
Hypoxia induction of endothelial cell migration requiring G6PI and RASFs. (**A**) Hypoxia induced cell migration of HDMECs assayed by trans-well invasion chambers. (**B**) G6PI-siRNA suppressed the cell migration of HDMECs under hypoxia condition. (**C**) Cell chemotaxis of HDMECs by Boyden Chamber trans-well assays indicating the increased cell migration of HDMECs by RASF-conditioned medium. As a positive control for chemotaxis, recombinant human VEGF protein was added into the medium. (**D**) HDMECs chemotaxis toward G6PI-siRNA or control-siRNA treated RASF-conditioned medium indicating the requirement of G6PI expression in RASFs to attract endothelial cell migration. Data were presented as mean ± SEM(n = 3). *P < 0.05.

In order to further validate the effect of hypoxia and G6PI on angiogenesis in endothelial cell, cell migration assays were performed with HDMECs under conditions of normoxia and 3% O_2_ of hypoxia with or without the expression of G6PI. Hypoxia induced endothelial cell migration (Fig. 4A), which required the expression of G6PI (Fig. 4B).

Endothelial cell chemotaxis is an initial step during angiogenesis. Boyden Chamber trans-well assays were applied to determine the chemotactic response to RASFs of HDMECs. As a positive control for chemotaxis, recombinant human VEGF protein was added into the medium of low chamber. As shown in Fig. 4C, RASF-conditioned medium was able to attract the migration of HDMECs. Moreover, the conditioned medium from G6PI-expressing RASFs attracted endothelial cell migration much stronger than that from G6PI siRNA treated RASFs (Fig. 4D).

### G6PI regulates VEGF secretion from RASFs

**Figure 5:**
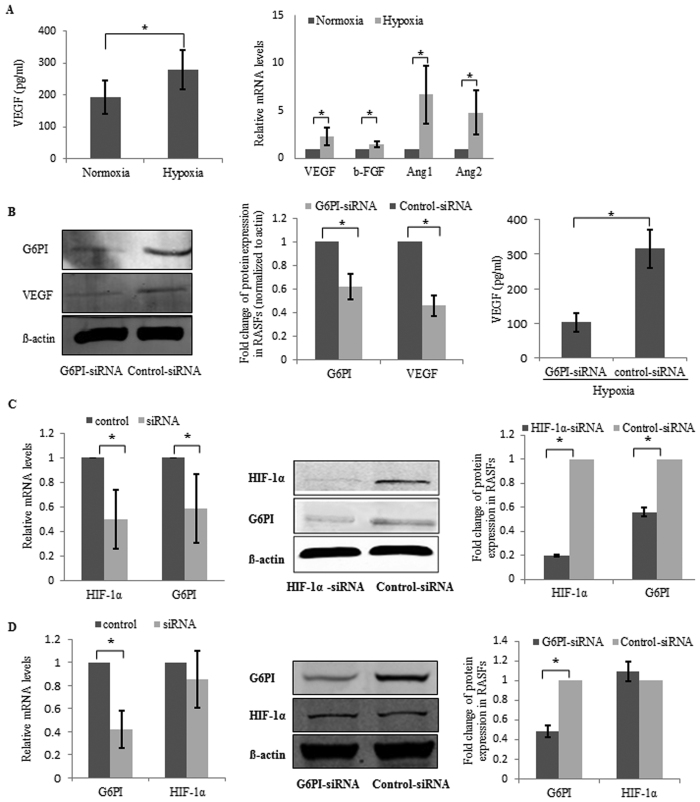
Mechanism study for the roles of G6PI plays during the regulation of hypoxia-induced angiogenesis. (**A**) Supernatant of RASFs was collected and assayed for VEGF abundance by ELISA demonstrating the induced VEGF secretion from RASFs by Hypoxia (left). In addition, hypoxia increased the mRNA expression of VEGF, β-FGF, Ang1 and Ang2 in RASFs (right). (**B**) Decreased expression of VEGF in RASFs by treatment with G6PI-siRNA at the protein level (left and middle). Decreased VEGF secretion from RASFs by treatment with G6PI-siRNA (right). (**C**) HIF-1α-siRNA inhibited the expression of G6PI in RASFs. (**D**) G6PI-siRNA did not affect the expression of HIF-1α in RASFs. Data were presented as mean ± SEM(n = 3). *P < 0.05.

In terms of the key role VEGF plays during angiogenesis, the concentration of VEGF in the medium of RASFs was determined by ELISA. Hypoxia induced VEGF secretion from RASFs (Fig. 5A). Angiogenesis-related growth factors, including VEGF, β-FGF (fibroblast growth factor), Ang1 and Ang2, showed increased mRNA levels in RASFs under hypoxia condition (Fig. 5B). In order to examine the relationship between G6PI and VEGF, G6PI siRNA was transfected into RASFs followed the analysis of mRNA and protein levels. VEGF expression was downregulated by G6PI siRNA (Fig. 5B). Accordingly, the level of secreted VEGF decreased after treatment with G6PI siRNA in RASFs (Fig. 5B).

### HIF-1α is an upstream regulator of G6PI in hypoxia condition

Since hypoxia induced G6PI expression, as well as HIF-1α expression, we further examined the relationship between HIF-1α and G6PI. Interestingly, knockdown of HIF-1α in RASFs deceased G6PI expression significantly (Fig. 5C), while knockdown of G6PI in RASFs did not affect the expression of HIF-1α (Fig. 5D). HIF-1α, as an upstream regulator of G6PI, may mediate the upregulation of G6PI by hypoxia in RASFs.

## Discussion

Synovitis is a basic pathological feature of RA. Synovial hyperplasia is always accompanied with infiltration of many inflammatory cells and release of inflammatory factors, leading to the formation of new blood vessels and pannus^25^. Pannus have similar erosion characteristics like tumor tissue^26^,^27^. It has been confirmed that hypoxia presents in the joint microenvironment of RA, and plays a key role in regulating angiogenesis in RA^28^. Hypoxia alters cellular bioenergetics by inducing mitochondrial dysfunction and promoting a switch to glycolysis, thereby leads to abnormal angiogenesis^29^. Glycolytic activity is enhanced in the hypoxia microenvironment of synovial tissues in RA^30^. The anaerobic metabolism level is positively correlated with synovitis in RA synovium^31^.

In this study we found G6PI expression increased significantly in the synovial tissue of RA, and especially enriched surrounding capillaries. We demonstrated that hypoxia induced HIF-1α and G6PI expression in both RASFs and HDMECs. We further confirmed that hypoxia promotes angiogenic tube formation and cell migration of HDMECs, and increases the cell proliferation of RASFs as well. The expression of G6PI is required for all of these phenotypes induced by hypoxia. Although we are the first to demonstrate that G6PI plays an important role in VEGF secretion from RASFs and mediate the hypoxia-induced angiogenesis in RA, it is consistent with Funasaka’s report that hypoxia-inducible VEGF regulates the PGI (phosphoglucose isomerase) expression, thereby enhances cancer cell motility^32^.

Abnormal angiogenesis is one of the characteristic features in RA^33^. Angiogenesis is important during synovial hyperplasia and progressive bone destruction^11^. There are quite a few factors involved in the regulation of angiogenesis including VEGF and FGF, which activate endothelial cells through binding to receptors^34^,^35^. Matrix metalloproteinases (MMPs) degrades base membrane and promote endothelial migration and proliferation to form vascular tubules^25^. Based on the role of angiogenesis during pathogenesis of RA, inhibition of joint neovascularization may be more effective in controlling synovitis and joint destruction^33^,^36^.

In RA synovium, abnormal proliferation of synovial fibroblasts and excessive recruition of leukocytes lead to oxygen consumption in joints, resulting in HIF-1α accumulation and hypoxia condition^37^,^38^. It has been reported that HIF-1α upregulates VEGF by interacting with PPARγ (peroxisome-proliferator-activated receptor-γ) and PPARγ co-stimulatory factor PGC-1α^39^. Hypoxia induces vascular reconstruction through HIF-1α, PGC-1α and VEGF^40^. In this study, we demonstrated that G6PI is a novel proangiogenic factor under hypoxia condition in RA.

Our findings demonstrated that hypoxia-induced overexpression of G6PI in RASFs may be responsible for the increased proliferation of RASFs in RA. Many studies have confirmed that hypoxia promotes the proliferation of RASFs, which plays important role during the pathogenesis of RA^41^,^42^,^43^. We have previously found the G6PI overexpression in promoting cell proliferation in RASFs^21^. In addition, G6PI inhibited apoptosis in RASFs as a key member in glycolysis. A recent publication reported the reliance of RASFs on glucose metabolism, where the balance between glycolysis and oxidative phosphorylation was shifted toward glycolysis compared to OA synovial fibroblasts^44^.

**Figure 6:**
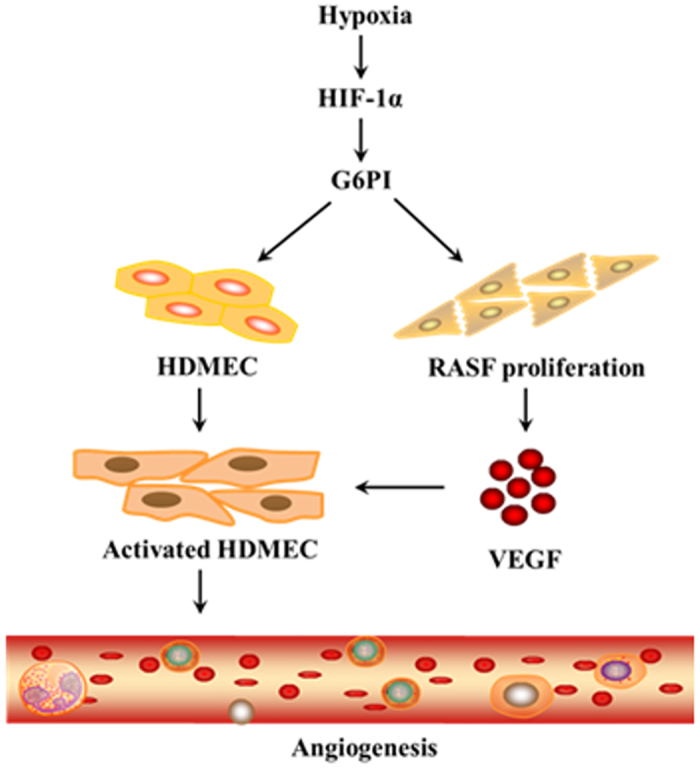
Schematic model for the role G6PI plays in regulation of hypoxia-induced angiogenesis in RA. This figure was drawn by ScienceSlides 2005 edition.

In summary, we demonstrated the increased VEGF secretion from RASFs partly mediated the hypoxia-induced angiogenesis in RA. In addition, hypoxia induces the G6PI and VEGF expression in HDMECs, which directly regulate the hypoxia-induced angiogenesis in RA. Taken together, we defined a novel pathway regulating hypoxia-induced angiogenesis in RA mediated by G6PI (Fig. 6).

## Materials and Methods

### Patient recruitment, arthroscopy and sample collection

Ten RA patients and ten osteoarthritis (OA) patients were recruited from Shanghai East Hospital. All the subjects fulfilled the 2010 American College of Rheumatology (ACR) criteria for the diagnosis of RA and OA. All patients were provided with written informed consent. The detailed protocol was approved by the Ethics Committee of Shanghai East Hospital (2012–df–043). Prior to tissue collection, signed informed consent was obtained from each patient. This study was conducted in accordance with the guidelines of the Declaration of Helsinki.

### Cells

Human dermal microvascular endothelial cells (HDMECs) were purchased from PromoCell and maintained in EBM endothelial basal medium supplemented with 5% fetal calf serum, 5 ng/ml human epidermal growth factor, 10 ng/ml basic fibroblast growth factor, 20 ng/ml insulin-like growth factor, 0.5 ng/ml vascular endothelial growth factor, 1 μg/ml ascorbic acid and 0.2 μg/ml hydrocortisone. Passages 3 to 8 cells were used for experiments. RA synovial fibroblasts (RASFs) were isolated from synovial biopsy by mincing into pieces of 2 to 3 mm and spreading on the bottom of cell culture flasks in RPMI 1640 medium (Life Technologies), supplemented with 10% fetal calf serum in a humidified atmosphere containing 5% CO_2_. RASFs were grown further over 4 to 8 passages. HDMECs and RASFs were cultured for 24 hours under normoxia condition and/or 3% O_2_ hypoxia condition in BioSpherix oxygen control system.

### Immunohistochemistry analysis

To determine the expression and distribution of G6PI in the synovium, immunohistochemical analysis was performed in RA (n = 10) and OA (n = 10) synovial tissue samples. The tissues were fixed in 10% neutral buffered formalin and embedded in paraffin and then cut into 5-um thick sections, de-paraffinized and rehydrated. The sections were heated at 95 °C for 20 minutes with Dako Target Rerieval solution (Dako, Copenhagen, Denmark) and incubated with primary antibodies against human G6PI mAb (1:500, Abcam) at 4 °C overnight. IgG control was used as negative control. HRP-conjugated secondary antibody was used as secondary antibody (Envision^TM^ Detection Kit, Dako) for 30 minutes at room temperature. Finally, diaminobenzidine (DAB) substrate kit was used to visualize the sections according to the manufacturer’s instructions. A semi-quantitative analysis was applied to the lining layer, sublining layer and vascular region using a well-established scoring method as described before^13^. The percentage of positive cells was assigned a score of 0 to 4, where 0 = no staining, 1 = 1–25% staining, 2 = 25–50% staining, 3 = 50–75% staining, and 4 = 75–100% staining.

### RNA extraction and quantitative reverse transcription-polymerase chain reaction (RT-PCR)

Total RNA was isolated from cells using TRIzol^TM^ (Invitrogen). Samples with a ratio of absorbance at 260/280 nm >1.8 were used, and total RNA was reverse transcribed to complementary DNA (TaKaRa) according to the manufacturer’s instructions. Gene expression was analyzed by relative quantification using Premix Ex Taq SYBR Green PCR (TaKaRa) on an ABI 7500 Real Time PCR System (Applied Biosystems). The primer sequences were as follow: G6PI Forward: 5′-AGG CTG CTG CCA CAT AAG GT-3′ and reverse 5′-AGC GTC GTG AGA GGT CAC TTG-3′; HIF-1α Forward: 5′-CCT ATG ACC TGC TTG GTG CT-3′and reverse: 5′-GCA AGC ATC CTG TAC TGT CCT-3′; VEGF Forward: 5′-CAC CCA CCC ACA TAC ATA CA-3′ and reverse: 5′-CTC AAG TCC ACA GCA GTC AA-3′; bFGF Forward: 5′- GAT TCA GTG GGT TGG GGG CA-3′ and reverse: 5′-AGG TCA GGT GAG GTT CGG GG-3′; Angiopoietin-1(Ang1) Forward: 5′-GGC AGT ACA ATG ACA GTT TC-3′ and reverse: 5′-CTT TGT TGC TTT CAT AAT CGC-3′; Angiopoietin-2(Ang2) Forward: 5′-CAG AGG CTG CAA GTG CTG GAG AAC A-3′ and reverse: 5′-GAG GGA GTG TTC CAA GAG CTG AAG T-3′. Expression of GAPDH was tested as an endogenous control for relative quantification and the primer sequences were as follows: forward 5′-GTG TCC AGC CTG AAT TCC ACT-3′and reverse 5′-CAC CCT GTT GCT GTA GCC AAA-3′. Results were analyzed using the ΔΔCt method for relative quantification and depicted as mRNA expression fold change relative to GAPDH.

### Western blot

RASFs and HDMECs were lysed in lysis buffer and centrifuged at 14,000 g for 5 minutes. The supernatant was run on sodium dodecyl sulfate-polyacrylamide gel electrophoresis followed by transferring onto nitrocellulose membranes (Amersham Pharmacia Biotech). Membranes were blocked with 5% nonfat dry milk for 1 hour at room temperature before incubated with mouse monoclonal anti-G6PI (1:500; Abcam), mouse monoclonal anti-HIF-1α(1:500; Abcam) or rabbit monoclonal anti-VEGF (1:200; Santa Cruze) at 4 °C overnight with gentle agitation. β-actin (1:1000; Santa Cruze) was used as a loading control. Following 3 × 15-minute washing in PBST buffer, membranes were incubated in DyLight 680-conjugated anti-mouse IgG or anti-rabbit IgG for 1 hour at room temperature. All immunoreactive proteins were visualized with ODYSSEY System and densitometric analysis of the bands was performed using Image J software.

### Cell proliferation assays

Cell proliferation was determined by MTT assays. Briefly, RASFs that had been transiently transfected with G6PI-siRNA or control-siRNA were plated at 1 × 10^3^ cells/well in 96-well plates and incubated under conditions of normoxia or 3% O_2_ of hypoxia. The CellTiter 96 Aqueous One Solution Cell Proliferation Assay Kit (Promega, Beijing, China) was used according to the manufacturer’s instructions. At the end of each period, MTT reagent was added to each well and incubated for 4 h. Then the formazan crystals were solubilized in DMSO and optical density (OD) value was read at 570 nm on a spectrophotometric plate reader. Each experiment was performed in triplicates.

### Cell cycle assays

Cell cycle was performed by flow cytometry analysis. After collecting cells and rinsing with cold PBS, and a total of 1 × 10^6^ cells were fixed with 70% ice-cold ethanol for 24 h at 4 °C. Cells were resuspended with cold PBS followed by incubation with 50 μg/ml propidium iodide and 0.1 mg/ml RNase A at 37 °C for 15 min. The DNA content of RASFs was acquired with BD FACS Calibur cytometry and analyzed by ModFitLT software.

### Gene silencing by RNA interference

Cells were seeded at a density of 1 × 10^5^ cells/well in 6-well plates maintained in complete growth medium until they were 60–70% confluent. 100 nM Gene-specific siRNA duplex against G6PI, HIF-1α or negative control and 5 μl Lipofectamine 2000 reagent in 990 μl serum/antibiotic-free Opti-MEM (Invitrogen) were mixed gently at room temperature for 20–30 minutes to form the complex. The mixed solution was overlaid on the cells for 4–6 hours before being replaced with complete medium supplemented with 10% FCS and antibiotic and incubated at 37 °C for 24 hours. The target sequences for G6PI-siRNA (5′-CCA TAC GGA AGG GTC TGC ATC ACA ATT-3′), HIF-1α-siRNA (5′-CAG GAC AGT ACA GGA TGC TTG CCA A-3′) and negative control-siRNA (5′-TTC TCC GAA CGT GTC ACG T-3′) were synthesized by GenepharmInc (Shanghai, China). The percent knockdown of G6PI expression was determined using quantitative RT-PCR and western blotting.

### Matrigel tube formation assay

Matrigel (50 μl, Corning) was polymerized for 1 hour at 37 °C in a 96-well plate. HDMECs that had been transiently transfected with G6PI-siRNA or control-siRNA were plated in 250 μl EBM/well on the surface of the Matrigel and incubated under conditions of normoxia or 3% O_2_ hypoxia for 24 hours. Assays for each condition were performed in triplicate. 5 randomly selected fields for each well were used to quantify tube formation by phase-contrast microscopy.

### Trans-well migration assay

HDMECs that had been transiently transfected with G6PI-siRNA or negative control-siRNA were plated in Transwell invasion chambers (Corning) on membranes precoated with Matrigel (Corning) containing EBM supplemented with 1% FCS, and EBM supplemented with 5% FCS in the lower wells. After 24 hours’ incubation under conditions of normoxia or hypoxia, Matrigel were removed with cotton swab, and the cells were fixed and stained with 0.1% crystal violet solution and assessed by two observers in a blinded manner. For the HDMECs chemotaxis assay, 10 nM VEGF-containing medium or RASFs-conditioned medium was used in the low chamber.

### Enzyme-linked immunosorbent assay (ELISA)

Human VEGF levels were measured in the supernatant of HDMECs and RASFs by using commercially available kits (R&D).

### Statistical analysis

All analyses were performed using SPSS 20.0 program package. Data are presented as mean ± SEM. Parametric Student’s t-tests were performed for the analysis of paired and unpaired samples. One-way analysis of variance (ANOVA) on ranks among three or more groups was performed. P values less than 0.05 were considered as significant.

## Additional Information

**How to cite this article**: Lu, Y. *et al*. Glucose-6-Phosphate Isomerase (G6PI) Mediates Hypoxia-Induced Angiogenesis in Rheumatoid Arthritis. *Sci. Rep.* **7**, 40274; doi: 10.1038/srep40274 (2017).

**Publisher's note:** Springer Nature remains neutral with regard to jurisdictional claims in published maps and institutional affiliations.
